# Surface Reconstruction of Perovskites for Water Oxidation: The Role of Initial Oxides’ Bulk Chemistry

**DOI:** 10.1002/smsc.202100048

**Published:** 2021-10-27

**Authors:** Haiyan Li, Yubo Chen, Justin Zhu Yeow Seow, Chuntai Liu, Adrian C. Fisher, Joel W. Ager, Zhichuan J. Xu

**Affiliations:** ^1^ School of Materials Science and Engineering Nanyang Technological University Singapore 639798 Singapore; ^2^ The Cambridge Centre for Advanced Research and Education in Singapore 1 CREATE Way Singapore 138602 Singapore; ^3^ Energy Research Institute@NTU ERI@N Interdisciplinary Graduate School Nanyang Technological University Singapore 639798 Singapore; ^4^ Key Laboratory of Materials Processing & Mold (Zhengzhou University) Ministry of Education Zhengzhou University Zhengzhou 450002 China; ^5^ Department of Chemical Engineering University of Cambridge Cambridge CB2 3RA UK; ^6^ Department of Materials Science and Engineering University of California at Berkeley Berkeley CA 94720 USA; ^7^ Berkeley Educational Alliance for Research in Singapore Ltd. 1 CREATE Way Singapore 138602 Singapore

**Keywords:** bulk chemistry, electrocatalysts, perovskite oxides, surface reconstruction of perovskites, water oxidation

## Abstract

Developing highly active electrocatalysts for oxygen evolution reaction (OER) is crucial for the scalable production of renewable hydrogen fuels by water electrolysis. Perovskite oxides are extensively studied as OER catalysts as they can have high activity and also offer considerable flexibility in composition and structure. Recently, there are increasingly numerous reports regarding dynamic surface reconstruction of perovskite oxides under OER conditions, with claims that the reconstruction‐derived species are the actual catalysts responsible for the measured OER activity. To enable rational design of perovskite oxides as precatalysts to generate actual active components in situ, gaining essential understanding of their reconstruction behaviors is crucial. This perspective discusses the roles of initial bulk chemistry in the surface evolution process of perovskite oxides during OER, including the dependency of surface stability on electronic structure of the precatalyst and the possibility of occurrence of lattice oxygen evolution reaction and cation leaching on the surface of a perovskite oxide precatalyst. It is reasonably argued that tailoring the bulk properties of perovskite precatalysts, such as electronic structure, crystallographic structure, and ion stoichiometry, can influence the occurrence of surface reconstruction and the formation of actual active surface species.

## Graphical abstract

Typical characteristics of the reconstruction over perovskite surface during water oxidation are introduced. The importance of initial perovskites’ bulk chemistry in influencing the surface reconstruction behavior is discussed in‐depth. The origins of the promoting effect of perovskite surface reconstruction on catalyzing water oxidation are summarized. Finally, challenges in designing perovskite oxides as precatalysts with controllable surface reconstruction behaviors are proposed.

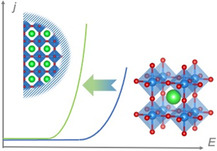

## 1 Introduction

Massive production of hydrogen gas through water splitting using electrical energy generated from renewable yet intermittent energy sources (such as sunlight, wind, and tides) could enable a future powered by a sustainable carbon‐free fuel.^[^ ^1^, ^2^ ^]^ The electrochemical reaction in deriving hydrogen from water is known as water electrolysis. According to the type of electrolyte applied, the water electrolysis can be classified into two categories of alkaline and proton‐exchange membrane (PEM) water electrolysis, both of which are promising for H_2_ production.^[^ ^3^ ^]^ Note that the electrolyzers using anion‐exchange membrane (AEM) is still under the development due to the challenges in membrane techniques. A brief comparison of the two water electrolysis technologies is shown in **Table** **1**.

**Table 1:** Comparison of alkaline and PEM water electrolysis technologies. Reproduced with permission.^[^ ^3^ ^]^ Copyright 2013, Elsevier

	Alkaline electrolysis	PEM electrolysis
Electrolyte	Caustic solution	Polymer electrolyte
Nominal current density	0.45 A cm^−2^	1.0 A cm^−2^
Energy consumption	4.35 kWh Nm^−3^ at 0.45 A cm^−2^	4.35 kWh Nm^−3^ at 1.0 A cm^−2^
Maximum current density	0.8 A cm^−2^	10 A cm^−2^
H_2_ delivery pressure	Up to 30 bars	Up to 700 bars
H_2_ purity (dry basis)	≥99.9%	≥99.99%
Lifetime	≥60 000 h	≥25 000 h
Dynamic range	0–100%	0–100%
Volumetric stack density	16 L Nm^−3^ h^−1^ H_2_	0.5 L Nm^−3^ h^−1^ H_2_

Basically, the water electrolysis consists of two half‐reactions, namely, hydrogen evolution reaction (HER) at the cathode and oxygen evolution reaction (OER) at the anode. In contrast with HER that involves two electron transfer steps, OER involves a more complex series of four electron transfer steps involving protons, contributing to a significant fraction of energy loss in water splitting.^[^ ^4^, ^5^ ^]^ The sluggish kinetics of OER can be further demonstrated with its much lower exchange current density (*i*_o,s_).^[^ ^4^ ^]^ For example, the *i*_o,s_ of IrO_2_ for OER is about 6 × 10^−7^ mA cm^−2^, while the *i*_o,s_ of Pt for HER can reach several hundreds of mA cm^−2^.^[^ ^6^, ^7^ ^]^ As a result, the high energy consumption of OER lowers the energy efficiency of the overall water electrolysis reaction and further impedes the commercialization of this hydrogen production technology. To address this issue, a wealth of research efforts has been made to develop efficient, robust, cost‐effective, and environmentally friendly electrocatalysts for OER.

Perovskite‐type oxides are an extensively studied oxide family for OER catalysis, owing to their great flexibility in chemical composition as well as the structure and therefore the exceptional adjustability of their physicochemical properties. The perovskite‐type oxides are generally classified into three main categories. The first category is the simple perovskite with the general formula ABO_3_ (where A and B are typically metal cations). An ideal perovskite oxide is in a highly symmetric cubic structure, with corner‐shared BO_6_ octahedrons constructing a 3D network and A‐site cations residing in the voids between octahedrons.^[^ ^8^ ^]^ Most of the metallic elements in the periodic table can be accommodated into the perovskite structure.^[^ ^9^ ^]^ Typically, A sites are occupied by relatively larger and more electropositive cations, for example, alkali, alkaline‐earth, and rare‐earth metals, while B sites are occupied by transition metals (TMs), which are smaller in radius and more electronegative, such as Co, Ni, Fe, Ir, and Ru. By partially substituting A‐ and/or B‐site cations with other elements, a greater number of perovskite compounds, described by the formula A_1−*x*_A′_*x*_B_1−*y*_B′_*y*_O_3_, can be created.^[^ ^10^ ^]^ In addition, the mismatch between the sum of positive charges of all cations and the sum of negative charges of oxygen anions can cause oxygen nonstoichiometry (in the form of either oxygen deficiency or oxygen excess), leading to a more general formula of A_1−*x*_A′_*x*_B_1−*y*_B′_y_O_3 ± *δ*_, where *δ* is a small real number describing the oxygen nonstoichiometry.^[^ ^10^ ^]^ The second category is double or layered perovskites. In these perovskites, the A‐site and/or B‐site are occupied alternately by different cations. The third category is Ruddlesden–Popper‐type oxide, which is expressed as A_*n *+ 1_B_*n*_O_3*n *+ 1_.^[^ ^11^ ^]^ This oxide consists of perovskite‐type ABO_3_ and rock salt‐type BO layers.

In the past decades, numerous efforts have been devoted to utilizing the perovskite oxide structure, especially the simple ABO_3_ perovskite, as a template to develop advanced OER electrocatalysts.^[^ ^12^, ^13^, ^14^, ^15^, ^16^, ^17^ ^]^ The observed superior activities of perovskite oxides once were directly correlated with their optimized bulk physicochemical properties. However, the latest studies have revealed the key role of surface reconstruction of some perovskites under OER conditions.^[^ ^18^, ^19^, ^20^, ^21^, ^22^, ^23^ ^]^ These works show that the reconstruction‐derived surface species, rather than the initial perovskite oxide, should be responsible for the measured activity,^[^ ^21^, ^22^, ^24^ ^]^ which is further experimentally supported by a recent work of our group.^[^ ^25^ ^]^ Nevertheless, for different starting materials, the dynamic structural and compositional responses with the variation of applied potential may be diverse,^[^ ^18^, ^21^, ^26^, ^27^, ^28^, ^29^, ^30^ ^]^ and the impact of surface changes on catalytic performance also varies.^[^ ^31^, ^32^, ^33^ ^]^ Therefore, the reconstruction behaviors and the ultimate OER activity are still expected to be strongly influenced by the bulk chemistry of initial materials (precatalysts).

For the rational design of perovskite oxides as the precursors for actual active components, it is vital to understand the relationships among the initial bulk chemistry, the surface evolution behavior, and the resulting catalytic performance. In this perspective, recent critical findings about the surface reconstruction of perovskites during water oxidation are summarized, and the roles of initial perovskites’ bulk chemistry in the reconstruction process are highlighted.

## 2 Typical Examples of Reconstruction over Perovskites’ Surface

**Figure 1:**
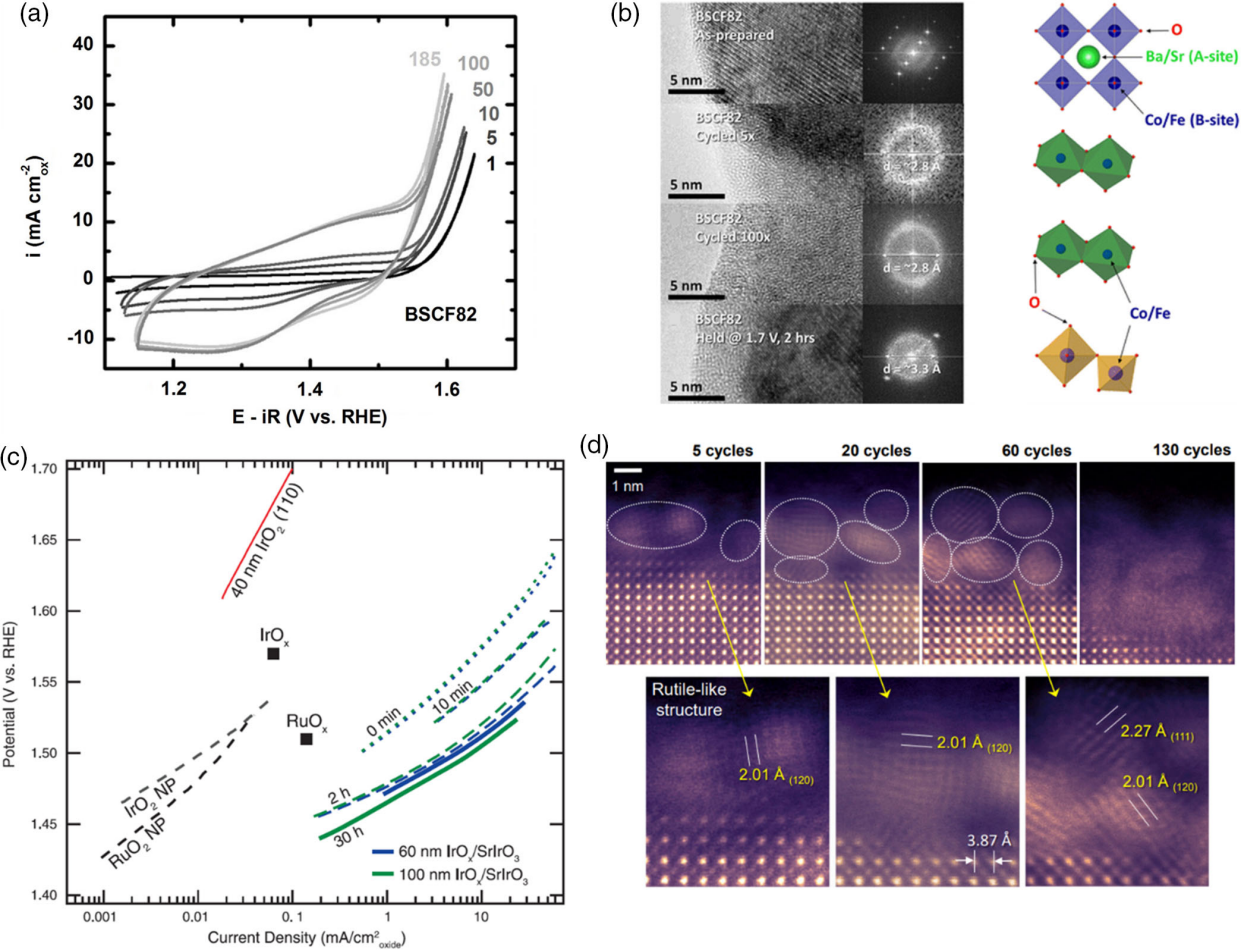
a) Cyclic voltammograms of BSCF (labeled as BSCF82) in 0.1 m KOH electrolyte, showing representative cycles. b) HRTEM images and corresponding FFT of BSCF particle surfaces before and after OER measurements. a,b) Reproduced with permission.^[^ ^18^ ^]^ Copyright 2012, American Chemical Society. c) Tafel plot comparing the specific activity of IrO_*x*_/SrIrO_3_ with other noble metal‐based OER catalysts in acidic electrolyte. Reproduced with permission.^[^ ^22^ ^]^ Copyright 2016, American Association for the Advancement of Science. d) High‐angle annular dark‐field images of pseudocubic SrIrO_3_ film surface layer derived from Sr leaching. Reproduced with permission.^[^ ^35^ ^]^ Copyright 2019, Elsevier.

A typical example of surface rearrangements of perovskite oxides during OER in an alkaline environment is that of Ba_0.5_Sr_0.5_Co_0.8_Fe_0.2_O_3−δ_ (BSCF). BSCF exhibited state‐of‐the‐art intrinsic OER activity (i.e., the OER current normalized by the oxide surface area) in 0.1 m KOH.^[^ ^12^ ^]^ The remarkably high activity of BSCF was initially attributed to the well‐optimized electronic configuration of its B‐site cations in the perovskite structure. This notion was later challenged when surface instability of BSCF under OER conditions was reported.^[^ ^18^, ^19^ ^]^ As shown in **Figure** **1**a, both the OER and the pseudocapacitive currents of BSCF increased considerately during potential cycling between 1.0 and 1.7 V versus RHE, suggesting that BSCF might have undergone surface changes induced by voltammetric cycling. As revealed by high‐resolution transmission electron microscope (HRTEM) imaging, the initially crystalline surface region of BSCF transformed into an amorphous layer with a thickness of ≈10 nm after five cycles, and the amorphized region thickened with further cycling (Figure 1b). In addition, the structural conversion was found to be accompanied by a significant loss of surface A‐site metals (i.e., barium and strontium). The fast Fourier transform (FFT) analysis of HRTEM images in Figure 1b disclosed formation of clusters consisting of edge‐sharing Co/Fe octahedrons in the cycled BSCF surface, in contrast to corner‐sharing octahedrons observed in the initial perovskite structure. Interestingly, for BSCF electrodes held at 1.7 V versus RHE for 2 h, spinel‐like structural motifs with Co/Fe cations in both octahedral and tetrahedral coordination were detected in the amorphized surface region (Figure 1b). It is noteworthy that the rapid amorphization of BSCF occurred when the applied voltage was above ≈1.5 V versus RHE, which is within the range of oxygen‐evolving potentials. When the applied potential was in the pseudocapacitive range, no visible surface transformation was observed.

In acidic environments, the promising candidates for OER electrocatalysts are limited to iridium‐based materials due to the relative thermodynamic stability of Ir.^[^ ^34^ ^]^ To reduce the utilization of the noble metal, accommodating Ir into complex oxides with inexpensive elements such as perovskites is a sensible approach.^[^ ^15^ ^]^ Most nonprecious metals in either A or B site typically will leach severely from perovskite oxides under acidic operation conditions, which inevitably brings about compositional and structural changes. Seitz et al. reported that highly active iridium oxide (IrO_*x*_), with an overpotential of only 270 mV at 10 mA cm^−2^_oxide_, could be derived from the surface layers of pseudocubic epitaxial SrIrO_3_ perovskite thin film (deposited on SrTiO_3_ (100) substrate through pulsed laser deposition [PLD]) via Sr leaching during electrocatalysis.^[^ ^22^ ^]^ It became increasingly active during 30 h of electrochemical testing in 0.5 m H_2_SO_4_ (Figure 1c) as a significant amount of Sr ions were significantly dissolved into the electrolyte. Density functional theory (DFT) calculations suggested that the experimentally observed activity improvement resulted from the in situ formation of structural motifs such as IrO_3_ or anatase IrO_2_, both of which resembled the initial pseudocubic perovskite in the arrangement of IrO_6_ octahedrons, on the Sr‐deficient surface. It was pointed out that the remaining SrIrO_3_ bulk played an indispensable role in the IrO_*x*_/SrIrO_3_ catalytic system by engendering a more active surface phase (IrO_*x*_). Through atomic‐scale direct probing, it was later identified that the leached surface of SrIrO_3_ films could evolve into either rutile‐like IrO_*x*_ nanocrystallites or amorphous states (Figure 1d).^[^ ^35^ ^]^

## 3 Role of Bulk Chemistry in Perovskites’ Surface Reconstruction

### 3.1 Occurrence of Surface Reconstruction

**Figure 2:**
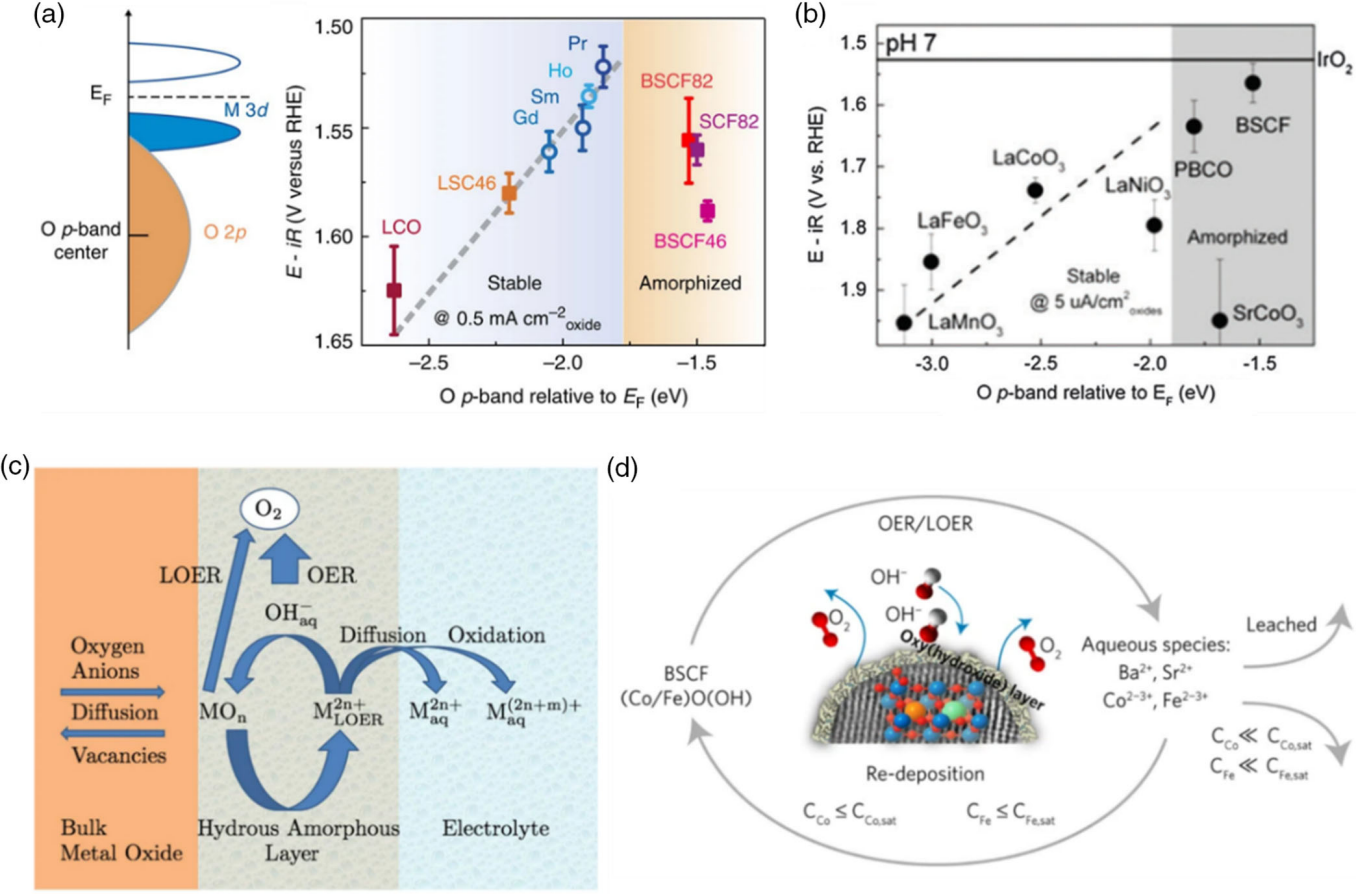
a) In the left panel, a schematic of the O *p*‐band for perovskite oxides; in the right panel, O *p*‐band center trend at pH 13 at 0.5 mA cm^−2^_oxide_ for LaCoO_3_ (LCO), La_0.4_Sr_0.6_CoO_3−*δ*_ (LSC46), (Ln_0.5_Ba_0.5_)CoO_3–*δ*_ with Ln = Pr, Sm, Gd, and Ho, BSCF (BSCF82), Ba_0.5_Sr_0.5_Co_0.4_Fe_0.6_O_3−*δ*_ (BSCF46), and SrCo_0.8_Fe_0.2_O_3−*δ*_ (SCF82). Reproduced with permission.^[^ ^17^ ^]^ Copyright 2013, Springer Nature. b) O *p*‐band center trend at pH 7 of selected oxides at 5 μA cm^−2^_oxide_. Reproduced with permission.^[^ ^47^ ^]^ Copyright 2015, Royal Society of Chemistry. c) Schematic representation of the OER, metal dissolution, and LOER processes as proposed by Binninger et al. Reproduced under the terms of the CC‐BY 4.0 license.^[^ ^48^ ^]^ Copyright 2015, The Authors, published by Springer Nature. d) Proposed mechanism for the self‐reconstruction of BSCF perovskite surface and formation of Co(Fe)O(OH) layer. Reproduced with permission.^[^ ^21^ ^]^ Copyright 2017, Springer Nature.

The transformation of the crystalline surface into an amorphous form during OER in alkaline media has also been reported for other earth‐abundant metal‐based perovskites, such as AFeO_3_ (A = Ca and Sr), La_2_NiMnO_6_, and La_2_Li_0.5_Ni_0.5_O_4_.^[^ ^36^, ^37^, ^38^, ^39^, ^40^, ^41^, ^42^, ^43^, ^44^, ^45^ ^]^ Interestingly, apart from amorphization, OER‐induced structural change can also occur through phase transformation. Lee et al. found that the surface region of BaNiO_3_ with a hexagonal perovskite structure was converted through exhaustive potential cycling into a new phase, BaNi_0.83_O_2.5_ (Ba_6_Ni_5_O_15_), that was stable against amorphization.^[^ ^46^ ^]^ However, some perovskites, such as La_0.4_Sr_0.6_CoO_3−δ_, LaCoO_3_, and LaMnO_3_, were found to be highly stable during OER given that little change was detected on their surfaces when they are subjected to electrochemical conditions that would have induced fast amorphization of BSCF.^[^ ^18^, ^19^ ^]^ To rationalize the difference in surface stability, an empirical theory was proposed by Shao‐Horn's group that having a bulk O *p*‐band center too close to the Fermi level (*E*_F_) potentially destabilizes the perovskite phase upon water oxidation in alkaline environments.^[^ ^17^ ^]^ As displayed on the right panel of **Figure** **2**a, for perovskites on the left branch, which have O *p*‐band centers lower than the threshold value of about −1.75 eV, crystalline surface regions were found to remain unchanged after OER measurements. In contrast, perovskites (on the right branch) having an O *p*‐band center as high as that of BSCF were found to undergo severe amorphization, along with the leaching of A‐site ions. In neutral electrolytes, two different ways of manifestation of surface instability were identified for perovskite‐type OER catalysts, which could be differentiated by the position of O *p*‐band center relative to *E*_F_ (Figure 2b).^[^ ^47^ ^]^ For perovskites with an O *p*‐band center far from *E*_F_, e.g., LaMO_3_ (M = Co, Ni, Mn, and Fe), the dissolution of catalytically active B‐site cations at high current or overpotential results in degradation, although their structural stability can be maintained at low current or overpotential. A high applied current density and overpotential can increase the thermodynamic driving force of B‐site ion oxidation and leaching.^[^ ^47^ ^]^ For example, the surface of LaCoO_3_ is stable at a low current density of $5{\text{ μA cm}}_{\text{ox}}^{-2}$ at pH 7, while reconstruction with B‐site Co leaching is observed at a high current density of $50{\text{μA cm}}_{\text{ox}}^{-2}$. In contrast, for perovskites with an O *p*‐band center close to *E*_F_, such as BSCF, SrCoO_3_, and PrBaCo_2_O_5 + δ_ (PBCO), surface amorphization accompanied by A‐site leaching takes place at both high and low overpotentials. This is related to the higher thermodynamic tendency of A‐site leaching. It is worth noting that the reconstruction behavior of a perovskite may be pH‐dependent, as LaCoO_3_, LaMnO_3,_ and PBCO are demonstrated to be stable catalysts at pH 13.^[^ ^17^, ^18^, ^47^ ^]^

From the perspective of thermodynamics, Schmidt's group demonstrated that regardless of the pH value, phase instability is unavoidable for any metal oxide under OER conditions owing to the oxidation of lattice oxygen anion through the lattice oxygen evolution reaction (LOER).^[^ ^48^ ^]^ In their work, three processes were considered, namely, chemical dissolution of the metal oxide, conventional OER, and LOER, and the chemical equilibria of these processes are interlinked. In the case of a metal oxide MO_*n*_ (*n* is a rational number) in alkaline conditions, the equations for the three processes can be written as follows.

(1) $$M^{2n+}O_{n}^{2-}+nH_{2}O↔M_{\text{aq}}^{2n+}+2n{\text{ OH}}_{\text{aq}}^{-}$$

(2) $$2{\text{ OH}}_{\text{aq}}^{-}↔\frac{1}{2}{\text{ O}}_{2}+H_{2}O+2e^{-}$$

(3) $$M^{2n+}O_{n}^{2-}↔M_{\text{aq}}^{2n+}+\frac{1}{2}n{\text{ O}}_{2}+2ne^{-}$$

From basic thermodynamic reasoning, it was concluded that for any type of metal oxide in aqueous electrolytes, the occurrence of LOER is thermodynamically favored at potentials above the oxygen evolution equilibrium potential. Consequently, the dissolved metal cations with high solubility can diffuse away from the electrode either with unchanged valency or in an oxidized state, which will cause the mass loss of the oxide electrode while those with extremely low solubility can recombine with aqueous oxygen anions and thereby participate in the LOER cation cycle, ultimately resulting in the formation of a hydrous surface layer with increased structural disorder (Figure 2c). The lattice oxygen vacancies created by LOER can be filled by hydroxide ions from the electrolyte (through recombination with the dissolved metal cations) or the lattice oxygen anions migrating from the bulk of the oxide.

Associating the aforementioned reasoning with the experimentally observed in situ formation of an oxy(hydroxide) layer on the BSCF surface, Schmidt's group proposed a mechanistic picture for the dynamic self‐reconstruction of BSCF upon the concurrence of OER and LOER (Figure 2d).^[^ ^21^ ^]^ While soluble A‐site cations, i.e., Ba^2+^ and Sr^2+^, leached from the perovskite, almost insoluble B‐site cations were redeposited on the oxide surface via the recombination with OH^−^ ions from the electrolyte, leading to the growth of a hydrous amorphous layer enriched with Co and Fe cations. Upon achieving a dynamically stable state, the Co/Fe oxy(hydroxide) surface layer coexisted with the underlying perovskite structure, and molecular oxygen was simultaneously evolved from the electrolyte and the oxy(hydroxide) lattice. Based on an operando X‐ray absorption spectroscopy (XAS) analysis of different Co‐based oxides, the same group identified the relationship between oxygen vacancy concentration and self‐reconstruction capability of metal oxides.^[^ ^21^ ^]^ In comparison with BSCF, less pronounced changes in the Co valency as well as the local atomic arrangements were observed for La_0.2_Sr_0.8_CoO_3–*δ*_ upon water oxidation, and even no change was detected for CoO. The oxygen vacancy content of the investigated catalysts varied in the same order as the extent of restructuring: BSCF > La_0.2_Sr_0.8_CoO_3−*δ*_ > CoO. Therefore, it was proposed that metal oxides rich in oxygen vacancies, such as BSCF, have the structural flexibility that allows drastic surface reconstruction during OER.

As LOER is an important driving force for the self‐reconstruction of metal oxides,^[^ ^48^ ^]^ physicochemical properties positively correlated with the ability of a metal oxide to undergo LOER can be intuitively linked to its reconstruction capability during OER. Both the presence of oxygen vacancies and the position of the O *p*‐band center (vs *E*_F_) have been reported to play crucial roles in facilitating the participation of lattice oxygen in the OER process over the surfaces of perovskites.^[^ ^14^, ^49^, ^50^, ^51^ ^]^ In addition, a linear relationship has been evidenced to exist between bulk oxygen vacancy formation energy and O *p*‐band center (vs *E*_F_) of perovskites.^[^ ^52^ ^]^ Therefore, a sufficiently small energy gap between the O *p*‐band center and the Fermi level, indicative of facile oxidation of lattice oxygen and formation of oxygen deficiency, is beneficial for the development of oxyhydroxide driven by LOER, which thus may explain the correlation found between the bulk electronic structure and the reconstruction behavior of perovskites in alkaline electrolytes (Figure 2a).

### 3.2 Formation of Actual Active Surface Sites

#### 3.2.1 Increase in the Numbers of Electrochemically Accessible Active Sites

With the increment of duration of potential cycling, the thickness of the amorphized region, the pseudocapacitive currents, and the OER currents of BSCF were found to simultaneously increase, which was attributed to the growth of porous amorphous layer with more active sites accessible to the electrolyte, leading to the enhancement of apparent catalytic performance.^[^ ^18^, ^19^ ^]^ Such a phenomenon has also been discovered on other perovskites during OER in alkaline electrolyte and was suggested to improve the observed OER activity in a way similar to that proposed for BSCF.^[^ ^36^, ^37^, ^41^ ^]^ For iridate perovskites in acid, the activity increment arising from dissolution‐induced surface roughening during potential cycling has also been reported.^[^ ^23^, ^33^, ^35^ ^]^ Therefore, it is reasonably expected that the reconstruction capability of a perovskite oxide during OER can significantly influence its apparent catalytic performance by increasing the electrochemically active surface area (ECSA). Note that the increment of ECSA due to surface reconstruction is limited as the pseudocapacitive currents and the OER currents always reach maxima.^[^ ^18^, ^22^, ^25^ ^]^ It signifies that the surface reconstruction of perovskite will finally reach a stable status after electrochemical activation.

#### 3.2.2 Formation of Active Metal Sites

As revealed by the HRTEM detection and the extended X‐ray absorption fine structure (EXAFS) analysis of cycled Ba_1−*x*_Sr_*x*_Co_0.8_Fe_0.2_O_3−*δ*_ (*x* = 0 and 0.5), the evolution of surface structures into amorphous states was accompanied by the conversion of corner‐sharing octahedrons into edge‐sharing octahedrons.^[^ ^18^, ^19^ ^]^ However, the contribution of local structural conversion to the measured OER activity remains elusive. Moreover, it is also unclear whether the structural motifs derived from different initial perovskites with the same active elements were identical and whether the intrinsic activities of these in situ developed active sites were comparable.

**Figure 3:**
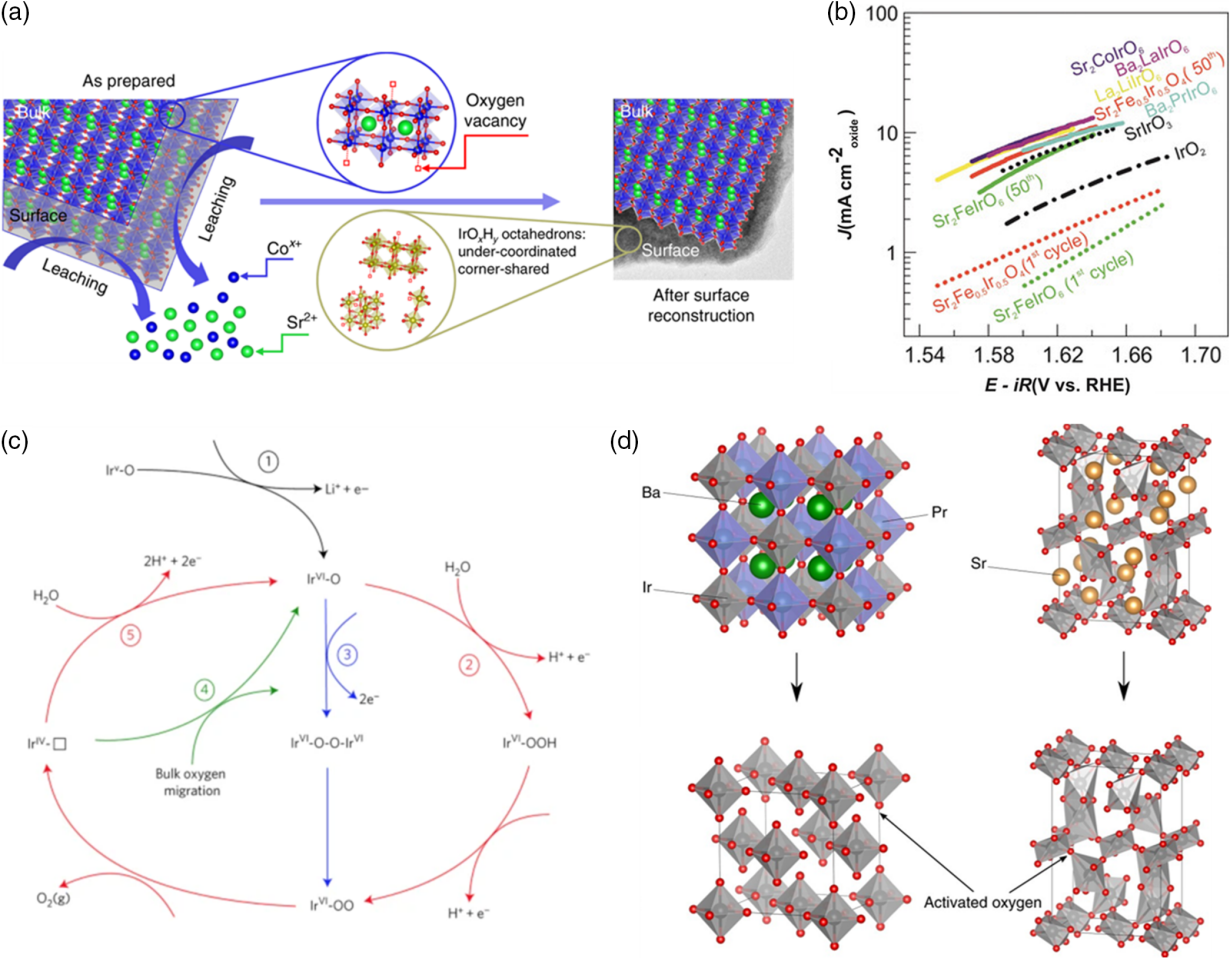
a) A schematic illustrating the surface reconstruction over SrCo_0.9_Ir_0.1_O_3‐δ_ surface. Reproduced under the terms of the CC‐BY 4.0 license.^[^ ^20^ ^]^ Copyright 2019, The Authors, published by Springer Nature. b) Tafel plots for the selected cycles of Ir‐based oxides. Reproduced with permission.^[^ ^24^ ^]^ Copyright 2019, Wiley‐VCH. c) Oxygen evolution mechanism on the surface of delithiated La_2_LiIr^(V)^O_6_ with oxidized surface oxygen as the reactive site. Reproduced with permission.^[^ ^53^ ^]^ Copyright 2017, Springer Nature. d) Formation of activated oxygen atoms on leached double perovskite (e.g., Ba_2_PrIrO_6_) and single perovskite m‐SrIrO_3_. Reproduced with permission.^[^ ^23^ ^]^ Copyright 2018, Springer Nature.

In the case of iridium‐based materials, some expect that the local structural arrangements in the resulting IrO_*x*_ phase are strongly correlated with those in the initial perovskite.^[^ ^22^, ^33^ ^]^ This was highlighted by a study of our group, in which exceptionally active iridium sites are derived from a pseudocubic SrCo_0.9_Ir_0.1_O_3−*δ*_ perovskite.^[^ ^20^ ^]^ The non‐noble metals in SrCo_0.9_Ir_0.1_O_3−*δ*_, i.e., Sr and Co, were found to leach out rapidly during OER in 0.1 m HClO_4_, which was expected to result in the collapse of surface perovskite structure and the formation of amorphous IrO_*x*_H_*y*_ surface layer, as shown in **Figure** **3**a. Based on a reasonable comparison of turnover frequency (TOF, i.e., amount of oxygen molecules produced per iridium site per second), iridium sites in the amorphized surface evolved from SrCo_0.9_Ir_0.1_O_3−*δ*_ were approximately a hundred times more active than those from monoclinic SrIrO_3_ (m‐SrIrO_3_) and over ten times more active than those from pseudocubic PLD‐SrIrO_3_ reported by Seitz et al.^[^ ^22^ ^]^ SrCo_0.9_Ir_0.1_O_3−*δ*_ as well as PLD‐SrIrO_3_ consisted of only corner‐sharing octahedrons, while m‐SrIrO_3_ was composed of face‐ and corner‐sharing IrO_6_ octahedrons. In addition, EXAFS analysis and DFT calculation collectively suggested that SrCo_0.9_Ir_0.1_O_3−δ_ is more oxygen‐deficient than m‐SrIrO_3_ and PLD‐SrIrO_3_, and therefore iridium ions within it were highly undercoordinated. Therefore, it was speculated that the corner‐sharing and under‐coordinated IrO_*x*_ octahedrons in the amorphous phase derived from SrCo_0.9_Ir_0.1_O_3−*δ*_ were intrinsically superior to the structural motifs evolved from m‐SrIrO_3_ and PLD‐SrIrO_3_.

Contrarily, Grimaud and coworkers proposed that the formation of amorphous IrO_*x*_ active phase in acid was resulted from the redeposition of dissolved iridium species onto the perovskite surface.^[^ ^24^ ^]^ As shown in Figure 3b, irrespective of the initial structure and composition, Ir^(V)^‐based perovskites, including Sr_2_MIrO_6_ (M = Fe, Co), Sr_2_Fe_0.5_Ir_0.5_O_4_, La_2_LiIrO_6_, and Ba_2_LnIrO_6_ (Ln = La, Pr), exhibited similar specific activities (i.e., OER activities normalized by the Brunauer–Emmett–Teller surface area of respective initial oxides) in 0.1 m HClO_4_. In addition, upon cycling in the acid electrolyte containing leached iridium ions, a blank glassy carbon electrode delivered gradually increasing OER currents. Accordingly, a reconstruction mechanism based on Ir dissolution–deposition equilibrium was put forward, that is, iridium dissolving from the perovskite could return to the catalyst surface via deposition as hydrated IrO_*x*_, the properties of which would determine the ultimate OER activity. The electronic structure of the initial perovskite, which controlled its open‐circuit potential (*E*_ocv_), would determine whether the iridium redeposition would be electrodeposition or precipitation. An *E*_ocv_ higher than the precipitation potential of IrO_*x*_ (*E*_p‐IrO*x*_ = 0.92 V vs RHE) suggests ready deposition of IrO_*x*_ without applied voltage, yielding stable catalytic performance of iridates such as Sr_2_CoIrO_6_. In contrast, for iridates with *E*_ocv_ < *E*_p‐IrO*x*_, such as Sr_2_FeIrO_6_ and Sr_2_Fe_0.5_Ir_0.5_O_4_, the growth of IrO_*x*_ phase via electrodeposition resulted in an electrochemical activation of the catalyst during anodic cycling.

#### 3.2.3 Formation of Electrophilic Oxygen Sites

Using a case study on La_2_LiIrO_6_ double perovskite, Grimaud et al. proposed that surface oxidation, e.g., in situ lithium removal, can result in the formation of electrophilic oxygen reactive sites, which accounts for surface instability as well as high activity of iridates.^[^ ^53^ ^]^ They found that La_2_LiIrO_6_ exhibited significantly higher OER activity in acid than in nonacid solutions. This was ascribed to in situ lithium extraction under acidic conditions where the applied anodic potential reached the delithiation potential measured in organic solvent (1.5 V vs NHE). After potential cycling at pH 1, crystalline IrO_2_ nanoparticles were detected on the surface of La_2_LiIrO_6_, along with surface depletion of La and Li, which was attributed to that delithiation had not only oxidized Ir^5+^ ions but also created vacancies for iridium ions to migrate from the bulk to the surface. DFT calculations demonstrated that the transition from La_2_LiIr^(V)^O_6_ to La_2_Ir^(VI)^O_6_ model phase would generate electron holes in some surface oxygen anions. Given the aforementioned observations, an OER mechanism involving the activation of surface oxygen sites was proposed (Figure 3c). The electrophilic character of surface oxygen anions activated by delithiation (step 1) could promote the formation of oxygen–oxygen bond, a critical rate‐determining step in the OER catalysis, via facilitating nucleophilic attack by a water molecule (step 2) or direct coupling of two neighboring oxygen radicals (step 3), thus enhancing the OER activity. The surface vacancies left by the release of oxygen molecules could be supplemented by the lattice oxygen migrating from the bulk (step 4) as well as the oxygen from water in the electrolyte (step 5). For La_2_LiIrO_6_, the conservation of crystalline bulk structure after potential cycling suggested that step 3 and step 4 played limited roles in OER catalysis.

Later, Geiger et al. suggested that the activated oxygen sites, i.e., the lower‐coordinated lattice oxygen anions (which was regarded as equivalent to the electrophilic O^I−^ species), can also be generated as the leached perovskites collapse into amorphous IrO_*x*_ in acid.^[^ ^23^ ^]^ It was experimentally demonstrated using online electrochemical mass spectrometry measurements of ^18^O‐labelled catalysts, in which the molecular dioxygen originated from lattice oxygen of amorphous IrO_*x*_ was detected during OER. The participation of lattice oxygen in oxygen evolution was believed to not only promote OER but also increase the probability of iridium dissolution because the generation of ephemeral oxygen vacancies resulted in weakly bonded iridium. The extent of lattice oxygen participation in OER over leached perovskite surfaces was determined by the number of activated oxygen sites in the ultimate IrO_*x*_ structure. In comparison with simple perovskite m‐SrIrO_3_ with non‐noble A‐site cations leaching from the voids of IrO_6_ octahedral network, the intensive loss of B‐site (in addition to A‐site) elements from double perovskites A_2_BIrO_6_ (A = Ba, Sr; B = Nd, Pr, Y) destroyed the 3D framework of corner‐sharing octahedrons and thus was expected to create a larger number of accessible activated oxygen atoms (Figure 3d), which explains their higher Ir dissolution rate and better OER‐specific activity (which was estimated by normalizing the OER currents to the pseudocapacitive charge extracted from the Ir redox features in cyclic voltammetry).

#### 3.2.4 Loss of Catalytically Active Sites

It is worth noting that the in situ reconstruction of perovskites is not always beneficial for catalyzing OER. For example, the severe loss of Ru sites at oxidative potentials has been suggested to account for the material degradation as well as the quick deactivation of SrRuO_3_ (SRO) perovskite in aqueous environment.^[^ ^54^, ^55^, ^56^ ^]^ Chang et al. studied the catalytic behaviors of SRO single crystals in alkaline solution and identified that the surface instability, which is positively correlated with the OER activity, increases in the order of (001) < (110) < (111).^[^ ^55^ ^]^ All the three SRO single‐crystal thin films were subjected to complete loss in activity during the first anodic scan, which was ascribed to the dissolution of active sites triggered by the electrooxidation of stable but inactive Ru^4+^ to unstable but active Ru^*n* > 4+^. The degrees of instability of the three films were estimated according to the amount of Sr and Ru dissolving into the electrolyte and the variation in film thickness after OER. It was proposed that the potential‐induced dissolution of Ru led to the chemical dissolution of Sr and thus collectively caused the disintegration of SRO films. The dependence of instability and activity on crystal orientation was proposed when oxidation rate from Ru^4+^ to Ru^n > 4+^ was related to the density of defects on the exposed surface. Afterward, Kim et al. explained the rapid deactivation of SRO polycrystalline particles in either alkaline or acid electrolyte from the perspective of thermodynamic instability.^[^ ^56^ ^]^ In contrast to the decomposition process proposed by Chang et al.,^[^ ^55^ ^]^ DFT‐computed Pourbaix diagram predicted that the dissociation of SRO perovskite in aqueous media started with the chemical dissolution of Sr upon contact with the aqueous electrolyte and followed by the electrochemical oxidation of Ru^4+^O_2_ to highly soluble Ru^8+^O_2_ species. Therefore, it was suggested that the disintegration of Ru‐based perovskites under OER conditions could be prevented by increasing thermodynamic stability without sacrificing catalytic efficiency. This was supported by recent findings that the fast decay in OER activity arising from the decomposition of SRO could be alleviated by tuning the A‐site composition of the initial perovskite.^[^ ^57^, ^58^ ^]^

## 4 Conclusion and Outlook

**Figure 4:**
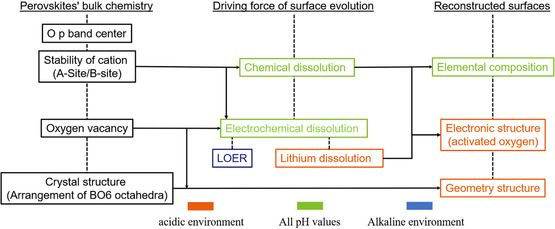
A schematic that illustrates the correlations among the specific features of initial bulk chemistry, the driving force of surface reconstruction, and the characters of reconstructed surfaces.

As shown in **Figure** **4**, the current understandings about the correlations among the specific features of initial bulk chemistry, the driving force of surface reconstruction, and the properties of reconstructed surfaces are summarized.

First, the bulk O *p*‐band center can be a reliable descriptor for perovskite surface stability in alkaline and neutral conditions and for the leaching behaviors of A/B‐site cations.^[^ ^17^, ^47^ ^]^ It, however, alone does not drive the surface reconstruction.

Second, the stability of A/B‐site cations can drive the perovskite surface reconstruction. Both chemical and electrochemical dissolution of cations have been observed. Chemical dissolution due to thermodynamic instability of cations is most common in all pH values. And then the elemental composition of the reconstruction surface is affected by the chemical dissolution. Particularly, the chemical dissolution in acidic environment also induces the formation of activated oxygen sites in the reconstructed surface.^[^ ^23^ ^]^ The electrochemical dissolution of Li cation was reported in La_2_LiIrO_6_ in acidic environment.^[^ ^53^ ^]^ After the Li dissolution, the reconstructed Li‐deficient La_2_IrO_6_ surface possesses active oxygen radicals.

Third, the oxygen vacancy content in initial perovskite bulk can indicate the tendency of electrochemical lattice oxygen dissolution, i.e., LOER.^[^ ^21^ ^]^ Note that the LOER has been demonstrated as a key driving force of surface reconstruction in alkaline environment and its possible effects on the reconstructed surface have rarely been considered. Nevertheless, for the surface reconstruction of Ir‐based perovskites in acidic environment, a high oxygen vacancy content in the initial bulk can induce the formation of undercoordinated active Ir‐sites in the reconstructed surface.^[^ ^20^ ^]^

Finally, the crystal structures of initial perovskite may not drive the surface reconstruction. However, the structural characters of initial perovskite, such as corner‐/edge‐shared octahedra, have been proposed to be inherited by the surfaces after reconstruction in acidic environment.^[^ ^20^, ^22^ ^]^

**Figure 5:**
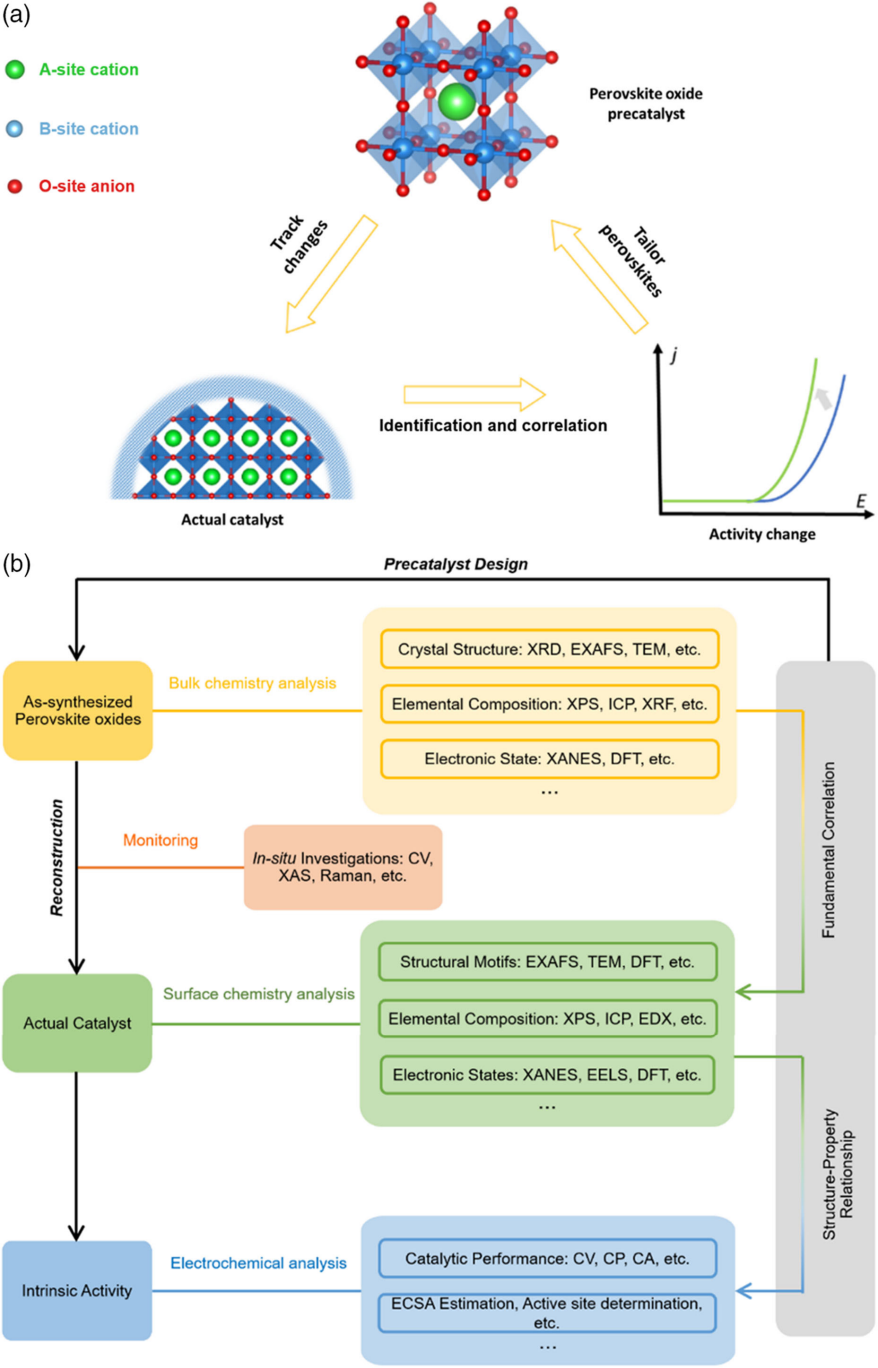
a) The general relation between precatalyst, actual catalyst, and catalytic activity change. b) The methodology for studying the reconstruction and understanding the related fundamentals. The tools for investigating the bulk chemistry, surface chemistry, and the reconstruction process: XPS, X‐ray photoelectron spectroscopy; ICP, inductively coupled plasma spectroscopy; XRF, X‐ray fluorescence spectroscopy; XANES, X‐ray absorption near‐edge structure; EDX, energy‐dispersive X‐ray spectroscopy; EELS, electron energy loss spectroscopy; CP, chronopotentiometry; CA, chronoamperometry.

Although reconstruction behaviors of perovskite oxides are subjected to the influence of initial bulk chemistry, it is complicated and the current knowledge is insufficient to establish design principles for the development of perovskite oxides as precatalysts (**Figure** **5**). Several critical issues pending future research are summarized as follows.

First, to understand how the reconstruction of perovskites influences catalytic performance, it is essential to determine the intrinsic activity of the actual catalyst. The restructuring of perovskites upon water oxidation is often accompanied by simultaneous changes in OER currents and ECSA. Therefore, it needs to be carefully examined whether the variation of measured OER kinetics is solely or cooperatively contributed by either the change in ECSA or the alteration in intrinsic activity. Turnover frequency (TOF), which reflects the inherent activity per active site, can be applied for activity evaluation if the number of reactive sites in the reconstructed surface can be reasonably estimated. Tentatively, all active elements, such as Fe, Co, Ni, and Ir, in the reconstructed surface regions can be considered to be the active sites.^[^ ^20^ ^]^ The TOF is then calculated with the equation of

(4) $$\text{TOF}=\frac{Q/\left(4\times e\right)}{\left(S\times d\right)/v\times n}$$

where *Q* is the total charge per second at a specific overpotential, $e=1.602\times 10^{-19}C$ is the charge of a single electron, *S* is the surface area of the catalyst, *d* is the depth of the reconstructed surface regions, *v* is the volume of the unit cell from initial crystallized perovskite, and *n* is the number of active elements in one unit cell. Then, the TOFs from a few representative OER catalysts are calculated and compared in **Table** **2**.

**Table 2:** The TOFs from representative perovskite‐type catalysts after surface reconstruction

Catalyst	TOF [s]^−1^	Overpotential [mV]	Electrolyte	Estimated depth of the reconstructed surface region	Refs.
Ba_0.5_Sr_0.5_Co_0.8_Fe_0.2_O_3−*δ*_	1.7	370	0.1 m KOH	8 nm	[18]
BaNiO_3_	22.3	370	0.1 m KOH	Outer (001) BaNi_0.83_O_2.5_ surface layer	[46]
SrCoO_3−*δ*_	0.044	370	0.1 m KOH	10 nm	[25]
SrCo_0.8_Fe_0.2_O_3−*δ*_	0.31	370	0.1 m KOH	10 nm	
CaCu_3_Fe_4_O_12_	0.68	370	0.1 m KOH	5 nm	[43]
Sr_0.98_Co_0.97_Si_0.03_O_3−*δ*_	0.287	370	0.1 m KOH	10 nm	[45]
La_2_LiIrO_6_	3.27	270	H_2_SO_4_ (pH = 1)	Outer (001) surface layer with Li leaching	[53]
SrIrO_3_‐thin film	0.20	270	0.5 m H_2_SO_4_	40 nm	[22]
SrIrO_3_‐monoclinic	0.03	270	0.1 m HClO_4_	5 nm	[20]
SrCo_0.9_Ir_0.1_O_3‐δ_	2.57	270	0.1 m HClO_4_	10 nm	

Second, to disclose the intrinsic structure–property relationship of the actual catalysts, it is necessary to precisely identify the local structural motifs of the reconstruction‐derived species. The reconstructed perovskite surface layers are typically in amorphous states, and their thickness can range from several to tens of nanometers. Due to the loss of long‐range order in the surface region and the potential interferences from the remaining bulk structure, it is challenging to directly probe the actual active components with traditional phase analysis tools, such as X‐ray diffraction (XRD) and TEM. Instead, surface‐sensitive characterization methods, such as X‐ray photoelectron spectroscopy (XPS), XAS in the electron yield mode, and low‐energy ion scattering spectroscopy (LEIS), are more suitable. In addition, operando characterization techniques can be helpful, as the unique characteristics of active sites in the reconstructed surface may only appear during the catalytic process. For example, in a recent study, the formation of a unique oxygen ligand environment during OER, i.e., the shrinkage of Ir—O metal ligand bonds, has been successfully detected in an amorphous IrO_*x*_ by operando XAS analysis.^[^ ^59^ ^]^ Moreover, DFT calculations can also aid in the atomic‐level exploration of the structural features that are critical to the OER catalysis.^[^ ^22^ ^]^

Third, to enable rational design of perovskite oxides as precatalysts with predictable reconstruction behaviors, it is necessary to further explore the fundamental relationships between initial bulk chemistry and surface evolution process. This is indispensable from comprehensive utilization of multiple experimental techniques to systematically investigate surface changes of various perovskites under electrochemical conditions.

Finally, when the principles behind the reconstruction behaviors of perovskites are elucidated, the next critical step is to adopt effective strategies to control surface evolution process by tailoring bulk properties of precatalysts for the development of desirable OER catalysts. For instance, the selective electrochemical etching of lattice cations, which may simultaneously introduce defects into the catalyst and enlarge its ECSA, has been demonstrated to promote the in situ derivation of highly active catalysts from perovskite hydroxides.^[^ ^60^, ^61^ ^]^

## Conflict of Interest

The authors declare no conflict of interest.

## References

[1] W. T. Hong , M. Risch , K. A. Stoerzinger , A. Grimaud , J. Suntivich , Y. Shao-Horn , Energy Environ. Sci. 2015, 8, 1404.

[2] Z. W. Seh , J. Kibsgaard , C. F. Dickens , I. Chorkendorff , J. K. Nørskov , T. F. Jaramillo , Science 2017, 355, eaad4998. 28082532 10.1126/science.aad4998

[3] P. Millet , S. Grigoriev , Renewable Hydrogen Technologies: Production, Purification, Storage, Applications and Safety, Elsevier B.V., Amsterdam, The Netherlands 2013, pp. 19–41.

[4] N.‐T. Suen , S.‐F. Hung , Q. Quan , N. Zhang , Y.‐J. Xu , H. M. Chen , Chem. Soc. Rev. 2017, 46, 337. 28083578 10.1039/c6cs00328a

[5] C. Wei , R. R. Rao , J. Peng , B. Huang , I. E. Stephens , M. Risch , Z. J. Xu , Y. Shao-Horn , Adv. Mater. 2019, 31, 1806296. 10.1002/adma.201806296 30656754

[6] K. Neyerlin , W. Gu , J. Jorne , H. A. Gasteiger , J. Electrochem. Soc. 2007, 154, B631.

[7] T. Schuler , T. Kimura , T. J. Schmidt , F. N. Büchi , Energy Environ. Sci. 2020, 13, 2153.

[8] M. Pena , J. Fierro , Chem. Rev. 2001, 101, 1981. 11710238 10.1021/cr980129f

[9] S. Royer , D. Duprez , F. Can , X. Courtois , C. Batiot-Dupeyrat , S. Laassiri , H. Alamdari , Chem. Rev. 2014, 114, 10292. 25253387 10.1021/cr500032a

[10] D. Chen , C. Chen , Z. M. Baiyee , Z. Shao , F. Ciucci , Chem. Rev. 2015, 115, 9869. 26367275 10.1021/acs.chemrev.5b00073

[11] P. Popper , S. Ruddlesden , Acta Crystallogr. 1957, 10, 538.

[12] J. Suntivich , K. J. May , H. A. Gasteiger , J. B. Goodenough , Y. Shao-Horn , Science 2011, 334, 1383. 22033519 10.1126/science.1212858

[13] Y. Zhu , W. Zhou , Z. G. Chen , Y. Chen , C. Su , M. O. Tadé , Z. Shao , Angew. Chem. 2015, 127, 3969. 10.1002/anie.201408998 25653050

[14] J. T. Mefford , X. Rong , A. M. Abakumov , W. G. Hardin , S. Dai , A. M. Kolpak , K. P. Johnston , K. J. Stevenson , Nat. Commun. 2016, 7, 11053. 27006166 10.1038/ncomms11053 PMC4814573

[15] O. Diaz-Morales , S. Raaijman , R. Kortlever , P. J. Kooyman , T. Wezendonk , J. Gascon , W. T. Fu , M. T. Koper , Nat. Commun. 2016, 7, 12363. 27498694 10.1038/ncomms12363 PMC4979062

[16] B. Zhao , L. Zhang , D. Zhen , S. Yoo , Y. Ding , D. Chen , Y. Chen , Q. Zhang , B. Doyle , X. Xiong , M. Liu , Nat. Commun. 2017, 8, 14586. 28240282 10.1038/ncomms14586 PMC5333368

[17] A. Grimaud , K. J. May , C. E. Carlton , Y.-L. Lee , M. Risch , W. T. Hong , J. Zhou , Y. Shao-Horn , Nat. Commun. 2013, 4, 2439. 24042731 10.1038/ncomms3439

[18] K. J. May , C. E. Carlton , K. A. Stoerzinger , M. Risch , J. Suntivich , Y.‐L. Lee , A. Grimaud , Y. Shao-Horn , J. Phys. Chem. Lett. 2012, 3, 3264.

[19] M. Risch , A. Grimaud , K. J. May , K. A. Stoerzinger , T. J. Chen , A. N. Mansour , Y. Shao-Horn , J. Phys. Chem. C 2013, 117, 8628.

[20] Y. Chen , H. Li , J. Wang , Y. Du , S. Xi , Y. Sun , M. Sherburne , J. W. Ager , A. C. Fisher , Z. J. Xu , Nat. Commun. 2019, 10, 572. 30718514 10.1038/s41467-019-08532-3 PMC6362036

[21] E. Fabbri , M. Nachtegaal , T. Binninger , X. Cheng , B.‐J. Kim , J. Durst , F. Bozza , T. Graule , R. Schäublin , L. Wiles , Nat. Mater. 2017, 16, 925. 28714982 10.1038/nmat4938

[22] L. C. Seitz , C. F. Dickens , K. Nishio , Y. Hikita , J. Montoya , A. Doyle , C. Kirk , A. Vojvodic , H. Y. Hwang , J. K. Norskov , Science 2016, 353, 1011. 27701108 10.1126/science.aaf5050

[23] S. Geiger , O. Kasian , M. Ledendecker , E. Pizzutilo , A. M. Mingers , W. T. Fu , O. Diaz-Morales , Z. Li , T. Oellers , L. Fruchter , Nat. Catal. 2018, 1, 508.

[24] R. Zhang , N. Dubouis , M. Ben Osman , W. Yin , M. T. Sougrati , D. A. D. Corte , D. Giaume , A. Grimaud , Angew. Chem. Int. Ed. 2019, 58, 4571. 10.1002/anie.201814075 30672081

[25] H. Li , Y. Chen , J. Ge , X. Liu , A. C. Fisher , M. P. Sherburne , J. W. Ager , Z. J. Xu , JACS Au 2021, 1, 108. 34467274 10.1021/jacsau.0c00022 PMC8395678

[26] Y. Zhu , W. Zhou , Y. Chen , J. Yu , M. Liu , Z. Shao , Adv. Mater. 2015, 27, 7150. 26450659 10.1002/adma.201503532

[27] Y. Duan , S. Sun , Y. Sun , S. Xi , X. Chi , Q. Zhang , X. Ren , J. Wang , S. J. H. Ong , Y. Du , L. Gu , A. Grimaud , Z. J. Xu , Adv. Mater. 2019, 31, 1807898. 10.1002/adma.201807898 30680800

[28] T. Wu , S. Sun , J. Song , S. Xi , Y. Du , B. Chen , W. A. Sasangka , H. Liao , C. L. Gan , G. G. Scherer , L. Zeng , H. Wang , H. Li , A. Grimaud , Z. J. Xu , Nat. Catal. 2019, 2, 763.

[29] X. Ren , C. Wei , Y. Sun , X. Liu , F. Meng , X. Meng , S. Sun , S. Xi , Y. Du , Z. Bi , G. Shang , A. C. Fisher , L. Gu , Z. J. Xu , Adv. Mater. 2020, 32, 2001292. 10.1002/adma.202001292 32567128

[30] Y. Duan , J. Y. Lee , S. Xi , Y. Sun , J. Ge , S. J. H. Ong , Y. Chen , S. Dou , F. Meng , C. Diao , A. Fisher , G. G. Scherer , X. Wang , A. Grimaud , Z. Xu , Angew. Chem. Int. Ed. 2020, 60, 7418. 10.1002/anie.202015060 33372346

[31] B. J. Kim , X. Cheng , D. F. Abbott , E. Fabbri , F. Bozza , T. Graule , I. E. Castelli , L. Wiles , N. Danilovic , K. E. Ayers , N. Marzari , T. J. Schmidt , Adv. Funct. Mater. 2018, 28, 1804355.

[32] B. J. Kim , E. Fabbri , D. F. Abbott , X. Cheng , A. H. Clark , M. Nachtegaal , M. Borlaf , I. E. Castelli , T. Graule , T. J. Schmidt , J. Am. Chem. Soc. 2019, 141, 5231. 30860837 10.1021/jacs.8b12101

[33] C. W. Song , J. Lim , H. B. Bae , S. Y. Chung , Energy Environ. Sci. 2020, 13, 4178.

[34] Z. J. Xu , Sci. China Mater. 2020, 63, 3. 10.1007/s40843-019-1261-1 PMC7094945 32219007

[35] C. W. Song , H. Suh , J. Bak , H. B. Bae , S. Y. Chung , Chem 2019, 5, 3243.

[36] A. Grimaud , C. E. Carlton , M. Risch , W. T. Hong , K. J. May , Y. Shao-Horn , J. Phys. Chem. C 2013, 117, 25926.

[37] Y. Zhu , W. Zhou , J. Sunarso , Y. Zhong , Z. Shao , Adv. Funct. Mater. 2016, 26, 5862.

[38] I. Yamada , H. Fujii , A. Takamatsu , H. Ikeno , K. Wada , H. Tsukasaki , S. Kawaguchi , S. Mori , S. Yagi , Adv. Mater. 2017, 29, 1603004. 10.1002/adma.201603004 27885701

[39] B. Hua , Y. F. Sun , M. Li , N. Yan , J. Chen , Y. Q. Zhang , Y. Zeng , B. Shalchi Amirkhiz , J. L. Luo , Chem. Mater. 2017, 29, 6228.

[40] Y. Tong , J. Wu , P. Chen , H. Liu , W. Chu , C. Wu , Y. Xie , J. Am. Chem. Soc. 2018, 140, 11165. 30132327 10.1021/jacs.8b06108

[41] B. Han , A. Grimaud , L. Giordano , W. T. Hong , O. Diaz-Morales , L. Yueh-Lin , J. Hwang , N. Charles , K. A. Stoerzinger , W. Yang , M. T. M. Koper , Y. Shao-Horn , J. Phys. Chem. C 2018, 122, 8445.

[42] D. Kowalski , H. Kiuchi , T. Motohashi , Y. Aoki , H. Habazaki , ACS Appl. Mater. Interfaces 2019, 11, 28823. 31339683 10.1021/acsami.9b06854

[43] S. Yagi , I. Yamada , H. Tsukasaki , A. Seno , M. Murakami , H. Fujii , H. Chen , N. Umezawa , H. Abe , N. Nishiyama , Nat. Commun. 2015, 6, 8249. 26354832 10.1038/ncomms9249 PMC4579779

[44] C. Yang , M. Batuk , Q. Jacquet , G. Rousse , W. Yin , L. Zhang , J. Hadermann , A. M. Abakumov , G. Cibin , A. Chadwick , ACS Energy Lett. 2018, 3, 2884.

[45] Y. Pan , X. Xu , Y. Zhong , L. Ge , Y. Chen , J.‐P. M. Veder , D. Guan , R. O'Hayre , M. Li , G. Wang , Nat. Commun. 2020, 11, 2002. 32332731 10.1038/s41467-020-15873-x PMC7181763

[46] J. G. Lee , J. Hwang , H. J. Hwang , O. S. Jeon , J. Jang , O. Kwon , Y. Lee , B. Han , Y.‐G. Shul , J. Am. Chem. Soc. 2016, 138, 3541. 26910187 10.1021/jacs.6b00036

[47] B. Han , M. Risch , Y.‐L. Lee , C. Ling , H. Jia , Y. Shao-Horn , Phys. Chem. Chem. Phys. 2015, 17, 22576. 26271910 10.1039/c5cp04248h

[48] T. Binninger , R. Mohamed , K. Waltar , E. Fabbri , P. Levecque , R. Kötz , T. J. Schmidt , Sci. Rep. 2015, 5, 12167. 26178185 10.1038/srep12167 PMC4503990

[49] X. Rong , J. Parolin , A. M. Kolpak , ACS Catal. 2016, 6, 1153.

[50] A. Grimaud , O. Diaz-Morales , B. Han , W. T. Hong , Y. L. Lee , L. Giordano , K. A. Stoerzinger , M. T. M. Koper , Y. Shao-Horn , Nat. Chem. 2017, 9, 457. 28430191 10.1038/nchem.2695

[51] J. S. Yoo , X. Rong , Y. Liu , A. M. Kolpak , ACS Catal. 2018, 8, 4628.

[52] Y. L. Lee , J. Kleis , J. Rossmeisl , Y. Shao-Horn , D. Morgan , Energy Environ. Sci. 2011, 4, 3966-.

[53] A. Grimaud , A. Demortière , M. Saubanère , W. Dachraoui , M. Duchamp , M.‐L. Doublet , J.‐M. Tarascon , Nat. Energy 2017, 2, 16189.

[54] S. H. Chang , J. G. Connell , N. Danilovic , R. Subbaraman , K. C. Chang , V. R. Stamenkovic , N. M. Markovic , Faraday Discuss. 2014, 176, 125. 25490237 10.1039/c4fd00134f

[55] S. H. Chang , N. Danilovic , K. C. Chang , R. Subbaraman , A. P. Paulikas , D. D. Fong , M. J. Highland , P. M. Baldo , V. R. Stamenkovic , J. W. Freeland , J. A. Eastman , N. M. Markovic , Nat. Commun. 2014, 5, 4191. 24939393 10.1038/ncomms5191

[56] B. J. Kim , D. F. Abbott , X. Cheng , E. Fabbri , M. Nachtegaal , F. Bozza , I. E. Castelli , D. Lebedev , R. Schäublin , C. Copéret , T. Graule , N. Marzari , T. J. Schmidt , ACS Catal. 2017, 7, 3245.

[57] S. Hirai , T. Ohno , R. Uemura , T. Maruyama , M. Furunaka , R. Fukunaga , W.‐T. Chen , H. Suzuki , T. Matsuda , S. Yagi , J. Mater. Chem. A 2019, 7, 15387.

[58] M. Retuerto , L. Pascual , F. Calle-Vallejo , P. Ferrer , D. Gianolio , A. G. Pereira , Á. García , J. Torrero , M. T. Fernández-Díaz , P. Bencok , M. A. Peña , J. L. G. Fierro , S. Rojas , Nat. Commun. 2019, 10, 2041. 31053713 10.1038/s41467-019-09791-w PMC6499887

[59] H. N. Nong , T. Reier , H.‐S. Oh , M. Gliech , P. Paciok , T. H. T. Vu , D. Teschner , M. Heggen , V. Petkov , R. Schlögl , T. Jones , P. Strasser , Nat. Catal. 2018, 1, 841.

[60] F. Song , K. Schenk , X. Hu , Energy Environ. Sci. 2016, 9, 473.

[61] B. Q. Li , Z. J. Xia , B. Zhang , C. Tang , H. F. Wang , Q. Zhang , Nat. Commun. 2017, 8, 934. 29038552 10.1038/s41467-017-01053-x PMC5643308

